# MASLD: Lipotoxicity and Imaging Parallels from Liver Steatosis to Kidney Injury

**DOI:** 10.3390/life15121805

**Published:** 2025-11-25

**Authors:** Sarmis Marian Săndulescu, Denisa Ștefania Ghiga, Diana Rodica Tudorașcu, Daniela Larisa Săndulescu, Adrian Mită, Marinela Cristiana Urhuț, Citto-Iulian Taisescu

**Affiliations:** 1 Department of Surgery, University of Medicine and Pharmacy of Craiova, 200349 Craiova, Romania; ssarmis@yahoo.com; 2 Doctoral School, University of Medicine and Pharmacy of Craiova, 200349 Craiova, Romania; 3 Department of Medical Semiology, University of Medicine and Pharmacy of Craiova, 200349 Craiova, Romania; petridiana@yahoo.com (D.R.T.); larisasandulescu@yahoo.com (D.L.S.); adrian.mita@umfcv.ro (A.M.); 4 Department of Internal Medicine, Emergency County Hospital of Craiova, 200642 Craiova, Romania; cristiana.urhut@yahoo.com; 5 Department of Physiology, University of Medicine and Pharmacy of Craiova, 200349 Craiova, Romania; citto.taisescu@umfcv.ro

**Keywords:** metabolic dysfunction-associated steatotic liver disease (MASLD), chronic kidney disease (CKD), lipotoxicity, Kidney Injury Molecule-1 (KIM-1), tubular injury biomarkers, hepatokines, controlled attenuation parameter (CAP), ultrasound-derived fat fraction (UDFF)

## Abstract

Metabolic dysfunction-associated steatotic liver disease (MASLD) is recognized as a systemic condition that is associated with an increased prevalence of chronic kidney disease (CKD), independent of classical risk factors. This review explores MASLD and metabolic kidney dysfunction, emphasizing lipotoxicity, emerging biomarkers, and liver–kidney fat imaging techniques. Renal fat is discussed as an ectopic lipid depot that may contribute to kidney vulnerability in the same cardiometabolic milieu as MASLD. In this context, lipotoxicity, a phenomenon intensively studied in MASLD, can affect multiple nephron segments, promoting fibrosis and, ultimately, CKD. Hepatokines may support the concept of a liver–kidney metabolic axis, but human data remain limited. Tubular biomarkers show promise for detecting early renal injury, but lack validation in large populations. Hepatic steatosis is quantified through multiple validated imaging techniques such as ultrasound, elastography, and magnetic resonance imaging (MRI). In contrast, renal fat imaging studies are limited and heterogeneous, and still lack standardization. In MASLD, an integrated hepatorenal assessment is warranted to capture the full burden of the disease.

## 1. Introduction: Interconnected Epidemics of MASLD and Renal Dysfunction

Chronic metabolic diseases represent a major and rising health challenge.

Metabolic dysfunction-associated steatotic liver disease (MASLD) currently affects nearly 30% of the adult population, with a prevalence exceeding 40% in certain regions, such as South America [1]. Forecasts indicate a continued increase by 2050, driven by the ongoing epidemics of obesity and insulin resistance (IR), with a change of the phenotypes toward higher rates of advanced fibrosis [2], possibly reflecting earlier onset of the disease [3].

It is well documented that MASLD is associated with an increased risk of cardiovascular events, obstructive sleep apnea, extrahepatic cancers, and renal dysfunction. In this context, the 2024 EASL-EASD-EASO Clinical Practice Guidelines recommend that patients with MASLD undergo routine assessment of kidney function, highlighting the systemic nature of MASLD and the clinical importance of the hepatorenal connection [4]. Similarly, data from the Global Burden of Disease indicate that chronic kidney disease (CKD) has a global prevalence of approximately 10%, with rates increasing alongside diabetes, hypertension, and obesity, mirroring MASLD trends [5,6].

MASLD is defined as hepatic steatosis with at least one cardiometabolic risk factor, such as overweight or obesity, type 2 diabetes (T2DM), or metabolic dysregulation [7]. Considering that this definition incorporates metabolic risk factors, it may be more useful in identifying individuals at risk of developing CKD than definitions based solely on imaging-detected steatosis [8].

CKD is defined as structural or functional abnormalities persisting for at least 3 months, including either a sustained estimated glomerular filtration rate (eGFR) < 60 mL/min/1.73 m^2^ or markers of kidney damage detected by urine tests, imaging, or biopsy [9,10].

Recent studies report associations between MASLD and CKD even in the absence of classical risk factors such as diabetes and hypertension, supporting this clinical link [11]. These conditions share pathophysiological drivers, including genetic predisposition, gut dysbiosis, IR, lipotoxicity, and chronic inflammation, collectively consistent with this association [12,13,14,15,16]. In this context, markers reflecting IR have been explored in populations with metabolic dysfunction. These markers may help to identify individuals at higher cardiometabolic risk, underscoring IR as a central component in the pathophysiology shared by MASLD and CKD [17].

Moreover, CKD is not just a consequence of metabolic dysfunction. It can also exacerbate metabolic imbalance through atherogenic dyslipidemia, hypertension, increased IR, elevated uric acid levels, and inflammation [18,19]. Clinical and experimental data suggest that ectopic renal lipid accumulation with fatty acid-induced toxicity represents an under-recognized form of organ-specific lipotoxicity [20].

In this review, we use the term “fatty kidney phenotype” to describe an imaging-based pattern of ectopic renal fat, not a formal diagnosis. It reflects ectopic lipid deposition in and around the kidney—notably perirenal and renal sinus fat (RSF) and, in research settings, parenchymal fat estimated by chemical-shift magnetic resonance imaging (MRI). At present, to our knowledge, there are no universally accepted diagnostic criteria, standardized protocols, or clinical cut-offs for renal fat in humans. Accordingly, we avoid nosological overstatement and use this imaging pattern purely as a descriptive construct. It is thought to reflect a structural substrate for renal lipotoxic injury, although lipotoxic damage may occur even when imaging-visible fat is modest. This review explores the link between MASLD and renal metabolic dysfunction, with particular attention to lipotoxicity as a shared pathogenic mechanism, and discusses the potential role of hepatokines, biomarkers, imaging techniques, and clinical implications.

## 2. Search Strategy

We conducted a narrative review. PubMed/MEDLINE, Scopus, and Web of Science were searched for peer-reviewed, English-language articles through July 2025, combining subject headings and free-text terms with Boolean operators.

We included human and animal clinical studies (cohort, case–control, cross-sectional, randomized), meta-analyses/reviews, and major guidelines/consensus papers relevant to MASLD–CKD and lipid-driven injury. We excluded case reports and conference abstracts without full text. We prioritized larger or longitudinal cohorts and studies adjusting for obesity, glycemia, and blood pressure. Key mechanistic studies were consulted when clarifying pathways.

Two authors screened titles/abstracts and reviewed full texts as needed; disagreements were resolved by discussion. Reference lists and forward citations were hand-searched; duplicates were removed.

Search keywords: MASLD/Non-alcoholic fatty liver disease (NAFLD); CKD (albuminuria, eGFR); renal fat; lipotoxicity; lipidomics, hepatokines, fetuin-A, Fibroblast Growth Factor 21 (FGF21), Kidney Injury Molecule-1 (KIM-1); Neutrophil Gelatinase-Associated Lipocalin (NGAL); ultrasound; controlled attenuation parameter (CAP), MRI.

Given study heterogeneity in designs, populations, exposures, and outcomes, we did not undertake a quantitative synthesis.

## 3. Kidney Involvement in MASLD: Clinical and Epidemiological

This section synthesizes human clinical and epidemiological evidence linking MASLD with renal dysfunction. The steatotic liver actively propagates extrahepatic injury through alterations in lipid metabolism, IR, dysregulated hepatokines signaling, and chronic low-grade inflammation [21,22].

The kidney is exposed to the same pathogenic processes operating in the liver, driven by shared cardiometabolic exposures [23]. Dysbiosis is often considered a contributor, but studies have revealed distinct microbial profiles in MASLD and in CKD [24,25]. Beyond shared risk factors, MASLD and CKD also appear to share genetic susceptibility. In particular, variants of the patatin-like phospholipase domain-containing 3 (PNPLA3) gene have been identified in both liver involvement and kidney dysfunction [14,26]. These converging susceptibilities translate into measurable renal risk in population studies.

Large population cohorts and meta-analyses consistently support a MASLD-CKD link. In a nationwide Korean cohort of 214,145 adults, MASLD predicted incident CKD and albuminuria, with the MASLD subgroup showing the highest risk [27]. In another large cohort of 211,992 individuals, MASLD was associated with CKD, and renal risk increased cumulatively with the number of cardiometabolic risk factors [28].

Beyond single cohorts, a comprehensive meta-analysis by Mantovani et al. of 13 prospective studies (encompassing >1.2 million adults) demonstrated a 43% higher risk of developing stage ≥3 CKD independent of age, sex, obesity, hypertension, and baseline renal function. This association remained robust across diagnostic definitions, including imaging and histology [29].

A more recent meta-analysis by Liu et al., restricted to cohort design (8 studies, 9 cohorts), reported that MASLD is associated with a 38% higher risk of CKD. Associations persisted across body mass index (BMI) strata and sex and remained consistent irrespective of baseline CKD, diabetes, hypertension, or other cardiovascular disease [30].

Growing clinical evidence indicates that grades of liver steatosis and especially advanced fibrosis are associated with higher rates of CKD. Interestingly, individuals with severe steatosis, even after MASLD remission, still remain at a higher risk of developing CKD [31,32]. When interpreting the MASLD–kidney link, clinicians should consider non-metabolic causes of renal dysfunction as clinical confounders [33].

## 4. Lipotoxicity in MASLD and Renal Injury: Shared Metabolic Parallels

Building on these clinical patterns, we outline key mechanistic parallels between steatotic liver disease and renal lipotoxic injury. When adipose depots are saturated, lipid spillover may occur, leading to ectopic fat deposition in non-adipose tissues [34]. This accumulation of harmful lipids and their deleterious effects on organs not specialized for fat storage constitute a phenomenon known as lipotoxicity [35,36,37]. The liver and kidney are among the most energetically demanding and metabolically active organs, due to their involvement in processes like gluconeogenesis and detoxification, making them particularly vulnerable to lipotoxic damage [38,39]. While lipotoxicity is a well-established major driver of MASLD progression, similar processes are increasingly recognized in renal disease.

### 4.1. Hepatic Lipotoxicity in MASLD

**Figure 1:**
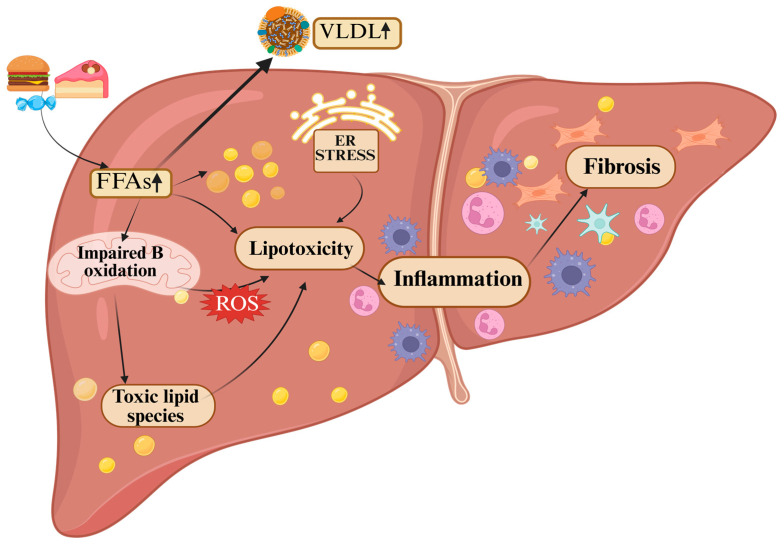
Mechanistic overview of hepatic lipotoxicity. When the liver’s buffering capacity is exceeded, excessive accumulation of toxic lipid species leads to mitochondrial dysfunction and increased production of reactive oxygen species (ROS), promoting endoplasmic reticulum (ER) stress and hepatocyte apoptosis, sustaining a pro-inflammatory microenvironment characterized by Kupffer cell activation and cytokine release, ultimately leading to fibrogenesis. Icons represent the mechanisms shown, arrows indicate the direction of the processes, and upward arrows denote increases. (Created in BioRender. Ghiga, D. (2025) https://BioRender.com/geows4c).

Under physiological conditions, the liver processes both dietary and endogenous lipids, acting as a metabolic sentinel. In MASLD, hepatocytes are overwhelmed by a chronic influx of free fatty acids (FFAs), and they enter a state of pathological compensation, in which triacylglycerol (TAG) storage serves as an initially protective buffer [40]. During transient rises in the availability of FFAs, the liver assembles TAG into very-low-density lipoproteins (VLDLs) for export, thereby preventing toxic lipid accumulation. Once these defense mechanisms are exceeded, lipotoxicity develops, triggering a cascade of cellular stress and injury mechanisms that drive the progression to inflammation and fibrosis [41], as summarized in Figure 1.

#### 4.1.1. CD36: The Inflammatory Entry Gate

Cluster of Differentiation 36 (CD36), a transmembrane fatty-acid translocase, is markedly upregulated in MASLD [42,43]. In the liver, this receptor is expressed in hepatocytes, Kupffer cells, hepatic stellate cells, and liver sinusoidal endothelial cells, facilitating lipid uptake, especially of long-chain fatty acids, and may also influence VLDL handling [44]. A mediator of oxidative stress is represented by oxidized low-density lipoprotein (ox-LDL) internalized by CD36 in Kupffer cells, where it leads to lysosomal stress and JNK (c-Jun N-terminal kinase) activation, contributing to inflammation in steatohepatitis [45]. In activated hepatic stellate cells, ox-LDL uptake via CD36 has been shown to stimulate the deposition of extracellular matrix (ECM) [46]. Garcia-Monzon et al. reported that the proteolytic cleavage of CD36 generates a soluble circulating form, which correlates positively with the severity of hepatic steatosis, suggesting biomarker potential [47]. Overall, the evidence suggests that CD36 should be regarded as both a transporter and a pathological inflammatory amplifier, serving as a conduit for toxic lipids and as a trigger for inflammation and immune dysfunction.

#### 4.1.2. Palmitate, Ceramides, and Diacylglycerols: Turning Fuel into Toxins

When a chronically sustained FFA delivery occurs, hepatocytes begin to accumulate toxic lipid species, such as saturated fatty acids, ceramides, lysophosphatidylcholines, and free cholesterol, which cannot be safely metabolized [39,48]. In a high-fat diet-fed mouse model, the inhibition of adipose triglyceride lipase was associated with improvements in IR and a reduction in hepatic steatosis [49].

Palmitate stands out among FFAs as a key driver of lipotoxicity. Its harmful effects arise from poorer incorporation into TAG compared with other unsaturated FFAs, leading to the accumulation of unesterified palmitate that stimulates inflammation and pro-apoptotic signals [50]. Also, saturated fatty acids are often diverted into the synthesis of molecules with profound cellular effects, such as ceramides and diacylglycerols (DAGs) [41].

Ceramides are sphingolipids whose toxicity depends on the fatty acid of origin. Palmitate-derived ceramides are regarded as mediators of hepatocellular injury through endoplasmic reticulum (ER) stress, impaired fatty acid β-oxidation, and IR [51].

#### 4.1.3. Organellar Breakdown

When lipids overload the hepatocytes, mitochondria face accelerated but inefficient β-oxidation [52], raising acetyl-CoA while generating excessive reducing equivalents, which overload the respiratory chain [53,54]. Electron leak generates reactive oxygen species (ROS) [55], which damages mitochondrial DNA and causes peroxidation of the inner mitochondrial membrane lipids, leading to loss of membrane potential [56].

In parallel, mitochondrial quality control shifts toward excessive fission, accumulating dysfunctional organelles that are suboptimally cleared [57]. Once mitochondrial damage exceeds the surveillance capacity, hepatocytes initiate the intrinsic apoptotic pathway through increased permeabilization of the mitochondrial outer membrane, the release of cytochrome c, and caspase activation [58].

ER homeostasis is perturbed by toxic lipid species, which disrupt calcium balance and membrane properties, triggering the unfolded protein response (UPR). Although this response is initially protective, chronic activation of the UPR promotes inflammation and hepatocyte apoptosis, contributing to disease progression [59].

Organellar failure drives the transition from intracellular stress to tissue-level inflammation and early matrix remodeling.

#### 4.1.4. Immune Escalation and the Death Spiral

Hepatic stellate cells activated by ROS or by damage-associated molecular patterns released during hepatocyte apoptosis transdifferentiate into myofibroblast-like cells. They synthesize ECM and connective tissue, promoting liver fibrosis [60].

The same signals activate Kupffer cells and monocyte-derived macrophages, which in turn activate NOD-like receptor protein 3 (NLRP3) inflammasome and secrete pro-inflammatory cytokines, including IL-1β, TNF-α, IL-6, and IL-18 [61,62,63]. Neutrophils are recruited to the liver by macrophage-derived chemokines. Once activated, they not only injure hepatocytes but also activate Kupffer cells, macrophages, and hepatic stellate cells by releasing granule proteins and neutrophil extracellular traps, thereby enhancing oxidative stress [63,64]. Beyond the innate cascade detailed above, adaptive immunity through dendritic cells, natural killer cells, and T and B lymphocytes also contributes, and its bidirectional crosstalk with innate immunity in MASLD has been recently reviewed [65].

Overall, the involvement of the immune system is described as a self-perpetuating inflammatory cycle, in which persistent activation maintains hepatocyte injury, driving fibrogenesis and progression from steatosis to steatohepatitis and ultimately to cirrhosis [63,66].

### 4.2. Renal Lipotoxicity: MASLD Parallels in Metabolic Injury

Moorhead et al. (1982) first postulated lipid-induced renal injury as a pathogenic mechanism of CKD progression, even though lipid accumulation in renal tissue had been observed decades earlier [67].

Under metabolic dysfunction, organs may become reservoirs for excess lipids. Whereas the liver is metabolically prepared to handle lipid overload, the kidney lacks such adaptive mechanisms [35]. Consequently, the renal cortex is particularly susceptible to lipid-induced injury due to its high metabolic demands and limited reliance on glycolysis, with oxidative metabolism predominating [20,68].

Experimental studies in murine models of high-fat diets have demonstrated renal lipid accumulation and glomerular injury, supporting a mechanistic link between dietary lipids and kidney damage [69]. Supporting this concept, Sucedaram et al. reported that rats fed a high-fat diet developed macro- and microvesicular steatosis with cellular ballooning. In parallel, in the kidneys, mesangial expansion and interstitial mononuclear inflammation were observed. In both organs, excessive infiltration of M1 macrophages was observed. These findings illustrate how diet-induced obesity simultaneously injures both organs via lipotoxic inflammation, consistent with a shared pathophysiology [70].

Consistent with these preclinical observations, human population studies with a similar diet (high-fat, high-sugar) have been associated with a high risk of developing CKD independent of diabetes [71]. The two major examples of CKD in which lipotoxicity is implicated are diabetic kidney disease (DKD) and obesity-related glomerulopathy (ORG) [72,73]. The latter is a histopathologic diagnosis (classically glomerulomegaly with focal segmental glomerulosclerosis-like patterns).

With this framework, we next outline nephron-segment-specific mechanisms of lipotoxicity.

#### Renal Lipotoxicity Across Nephron Segments

In a normal metabolic environment, the influx of FFAs into renal cells is mediated by several lipid transporters. Among them, CD36 is widely expressed in glomerular mesangial cells, podocytes, tubular epithelial cells, and interstitial macrophages [74].

Its expression is upregulated in diabetes and obesity-associated inflammation, particularly in tubular cells, driving lipid uptake beyond metabolic needs [75]. In experimental models, CD36-knockout diabetic mice exhibited improved mitochondrial function, a significant reduction in fibrosis, and decreased proteinuria, suggesting a pathogenic role for CD36 in promoting renal injury [76].

In the kidney, proximal tubular cells rely on β-oxidation as their main energy pathway [77]. Unlike hepatocytes, they lack an efficient bulk lipid export system, so when β-oxidation capacity declines, intracellular lipid accumulation leads to lipotoxicity and ultimately fibrotic remodeling [78].

Importantly, tubular cells are indirectly affected through glomerulo-tubular crosstalk secondary to glomerular damage. Podocyte disruption is central to this process, allowing for leakage of albumin and lipid-bound proteins into the filtrate [72].

Consequently, proximal tubular epithelial cells reabsorb albumin-bound non-esterified fatty acids and other lipids, accumulating intracellular fat. This process is a hallmark of nephron injury caused by metabolic disease and sustained by a continuous loop of glomerular leakage and tubular overload, ultimately leading to tubular fibrosis and progressive kidney dysfunction [79].

Podocytes are highly sensitive to lipid-mediated stress, in which cytoskeletal collapse leads to the effacement of their foot processes. Their apoptosis and detachment from the glomerular basement membrane contribute to proteinuria and progression of glomerular injury [72]. In uninephrectomized, high-fat-diet-fed mice, podocytes accumulate lipids, develop fragmented mitochondria, and activate autophagy, ultimately resulting in glomerular injury and secondary tubular stress. Lipidomic analysis in this model revealed the specific accumulation of cholesteryl esters, accompanied by decreased fatty acid β-oxidation, suggesting cholesterol-driven lipotoxic stress in podocytes [80].

Lipid accumulation in mesangial cells promotes their proliferation and activation, leading to increased ECM deposition. In obesity, this process contributes to the thickening of the glomerular basement membrane and drives glomerulosclerosis, ultimately leading to functional decline [75]. In addition, lipotoxicity triggers a local inflammatory response. FFAs stimulate tubular cells to release pro-inflammatory cytokines, which attract macrophage infiltration into the interstitium, thereby amplifying tubular and glomerular injury in a manner similar to immune activation seen in the steatotic liver [75].

## 5. Hepatorenal Biomarkers: From Liver Signals to Kidney Injury

This section summarizes circulating and urinary biomarkers that reflect metabolic stress and hepatorenal crosstalk, rather than lipid-specific signals alone—including mediators linked to lipid handling.

### 5.1. Biomarkers of Kidney Injury

Identifying non-invasive biomarkers that capture early lipid-induced kidney injury is important for detecting renal involvement in metabolic dysfunction as early as possible.

The most well-studied and established marker of renal dysfunction is albuminuria, which is included in the CKD definition [9]. Albuminuria can be found in obese or MASLD patients, even in the absence of hypertension or diabetes, supporting the idea that kidney dysfunction can be a consequence of lipid-induced stress [81,82].

New markers of renal injury, like Kidney Injury Molecule-1 (KIM-1) and Neutrophil Gelatinase-Associated Lipocalin (NGAL), reflect tubular epithelial damage and have been associated with both acute kidney injury (AKI) and CKD. Their value lies in their capacity to detect renal injury before the rise of classical biomarkers such as creatinine [83]. They respond to numerous stressors, including lipid overload. Urinary levels have been associated with early stages of DKD. Yet their role in non-diabetic metabolic kidney injury is still uncertain [84]. Goknar et al. reported in a small study of obese children without diabetes reported increased KIM-1 and N-acetyl-β-D-glucosaminidase (NAG) levels, independent of the IR status, suggesting early tubular stress occurs even in the absence of diabetes [84,85]. However, other pediatric cohorts did not confirm an association between urinary levels of KIM-1 or NGAL and BMI [86].

The urinary liver-type fatty acid-binding protein (uL-FABP) rises in early renal tubular injury, correlates with albuminuria, and precedes serum creatinine elevations. These features support earlier diagnosis and prognostic stratification in CKD and DKD [87,88].

Emerging multi-omics approaches (lipidomics, proteomics, metabolomics) are revealing novel biomarkers linking MASLD and CKD. In particular, renal lipidomics is beginning to reveal a reproducible circulating signature that complements eGFR and albuminuria. In diabetic cohorts, higher circulating lysophosphatidylethanolamines (LPEs), lysophosphatidylcholines (LPCs), phosphatidylethanolamines (PEs), phosphatidylcholines (PCs), and DAGs track the diabetes to the DKD continuum and correlate with more albuminuria and lower eGFR. A nine-lipid panel distinguished DKD from diabetes and, when combined with creatinine and urea, outperformed eGFR for early disease detection [89]. In CKD, a serum multimarker lipid panel predicted progression to ESKD independently of baseline eGFR and proteinuria, with similar performance in diabetic and non-diabetic subgroups and improved risk reclassification [90].

Taken together, these data support lipidomics as a complementary marker of kidney risk that needs to be tested prospectively in non-diabetic MASLD cohorts alongside eGFR and albuminuria. Looking ahead, integrating renal lipidomics with standardized renal-fat imaging and liver staging in MASLD cohorts may refine the characterization of MASLD-related kidney injury and support the development of outcome-anchored thresholds.

### 5.2. MASLD and Kidney Injury: Candidate Signals Behind the Parallels

In MASLD, the liver becomes a source of hepatokines, signaling molecules that contribute to immune activation and metabolic imbalance [91]. To our knowledge, only a few have been related to renal dysfunction, most notably Fetuin-A and FGF21.

#### 5.2.1. Fetuin A

Fetuin-A exerts numerous metabolic effects by inhibiting insulin receptors, impairing glucose transporter type 4 (GLUT4) translocation, and promoting de novo lipogenesis in hepatocytes. Additionally, it enhances FFA binding to toll-like receptor 4 (TLR4), thereby stimulating cytokine production and low-grade inflammation [92,93].

This glycoprotein has been widely studied in MASLD. Increased levels were observed in MASLD patients compared with healthy individuals. However, the results regarding their relationship with disease severity are controversial. A recent study found a positive correlation between steatosis grade, as measured by CAP. In contrast, a meta-analysis based on histologically confirmed MASLD cases did not demonstrate any association with metabolic dysfunction-associated steatohepatitis (MASH), and the findings regarding fibrosis were inconsistent [94,95]. Notably, TLR4 is also expressed in renal cells, where it is involved in inflammation and renal impairment by activating the immune system [96,97]. However, to our knowledge, there are no data supporting a direct interaction between fetuin-A and TLR 4 in the kidney.

The relationship between fetuin-A and kidney impairment is complex, involving both direct and indirect pathways. Ix et al. found no correlation between fetuin-A levels and renal function in a cohort of individuals with coronary artery disease [98]. In contrast, Bassey et al. demonstrated a direct link, with lower fetuin-A values associated with reduced eGFR, as well as an indirect association through BMI [99].

Fetuin-A concentrations are elevated in obese patients, where eGFR is also increased, indicating a link between fetuin-A and hyperfiltration, typically occurring in the early stages of CKD [99,100]. Moreover, obesity, diabetes, and metabolic syndrome are associated with both higher fetuin-A levels and CKD. For example, patients with obesity can develop proteinuria even at nephrotic levels [101,102]; mechanistically, it can be assumed that fetuin-A levels are also indirectly related to proteinuria or albuminuria.

It is worth noting that, while serum fetuin-A tends to decrease as CKD advances, urinary fetuin-A increases in parallel with worsening renal function, reflecting local kidney injury [103,104]. Higher urinary fetuin-A levels are linked to worse kidney outcomes and can predict progression to end-stage renal disease (ESKD) [105].

More recently, urinary proteomic studies have revealed that a peptide-containing fetuin-A fragment (uPTM-FetA) is a promising biomarker, which appears to be more sensitive than conventional markers such as albuminuria for DKD diagnosis [106,107].

#### 5.2.2. FGF21: A Stress-Induced Protector with Renal Relevance

The liver is the main source of FGF21, a protein recognized as a starvation or stress-induced hormone that exerts multiple biological roles across various tissues [108]. Among its metabolic functions, this hepatokine modulates macronutrient preferences, energy balance, enhances insulin sensitivity, promotes glucose uptake, and regulates hepatic triglyceride metabolism [109].

In the liver, FGF21 plays a crucial metabolic role by promoting fatty acid β-oxidation and insulin sensitivity, while limiting the secretion of VLDL and de novo lipogenesis. Through these actions, it protects against steatosis, lipotoxicity, oxidative stress, inflammation, and fibrosis [108,109].

More recently, this molecule has emerged as a biomarker in MASLD, with studies correlating it with inflammation and MASH severity [110,111]. Moreover, it has been suggested as a useful tool for prognosis and monitoring the response to treatment [112].

Despite its protective functions, FGF21 levels are found to be elevated in obese and diabetic patients [108]. However, considering that in both humans and animals the exogenous administration was shown to exert protective effects in obesity, liver steatosis, and even diabetes, a paradox has emerged. This has led to the conclusion that in metabolic disease, the cells experience FGF21 resistance, similar to IR [109,112,113].

Taking into account that MASLD does not occur in isolation and considering its common background with CKD, FGF21 has also been suggested to impact renal health. Serum FGF21 levels are increased in CKD and correlate with higher mortality [114,115]. Evidence indicates that these elevations are due to increased production in response to metabolic and inflammatory stress, not only to reduced clearance [116]. Experimental animal studies suggest a protective role of FGF21 on renal function, which involves reducing lipid accumulation, inflammation, oxidative stress, apoptosis, and even renal fibrosis [117,118,119].

Giontella et al. reported that genetically proxied higher levels of FGF21 are associated with increased eGFR, reduced proteinuria, and improved sodium excretion, thereby decreasing the risk of CKD. The study also found a correlation between FGF21 and more favorable cardiometabolic markers, such as fasting insulin, blood pressure, LDL cholesterol, and triglycerides [120].

Minami et al. showed that mice lacking FGF21 develop more severe kidney injury, along with autophagy stagnation, highlighting the potential role of FGF21 in preventing lipid-driven injury, which may be relevant in the context of MASLD [121].

To date, FGF21 has been primarily investigated separately in MASLD and renal dysfunction, particularly in diabetic cohorts. Further studies are needed to clarify its direct association with kidney outcomes in MASLD across both diabetic and non-diabetic populations.

To synthesize the hepatorenal parallels and markers discussed above, Table 1 maps the main lipotoxic modules with their liver and kidney read-outs, including a brief note on renal lipidomics.

## 6. Lessons from MASLD: Non-Invasive Imaging of Renal Fat

Emerging evidence suggests that ectopic lipid deposition in and around the kidney is clinically relevant, but imaging methods to assess these depots lag behind those for hepatic steatosis and remain unstandardized [122]. Interest in renal fat has arisen mainly from imaging studies showing it is not merely a benign finding, sometimes being associated with hypertension and CKD, as highlighted by the Framingham Heart Study [123].

B-mode ultrasound can detect liver steatosis using typical sonographic features [124]. By contrast, when evaluating a hyperechoic renal parenchyma, it usually reflects fibrosis in the context of CKD rather than fat deposition within the tissue [125]. While parenchymal fat cannot be evaluated by ultrasound, the thickness of the fat around the kidney can be measured. In their study, Lamacchia et al. reported that the thickness of the perirenal and pararenal fat, measured by ultrasonography, showed an inverse relationship with eGFR in T2DM patients [126].

For the liver, new quantitative ultrasound techniques, such as CAP and ultrasound-derived fat fraction (UDFF), have emerged for the non-invasive assessment of steatosis [127,128].

CAP is endorsed by the EASL guidelines and is already used in clinical practice. Reported thresholds for detecting hepatic steatosis vary slightly between studies, depending mainly on the reference standard and population characteristics, but most values for significant steatosis are above 275 dB/m [129]. UDFF is a promising technique that has shown strong correlation with magnetic-resonance-imaging-based proton density fat fraction (MRI-PDFF) in a recent multicenter study [128].

These techniques were developed and validated for the liver, and to date, there is no standardized or clinically validated application for the kidney.

Key liver and kidney fat imaging modalities are summarized in Table 2, aligning principle, assessable compartments, quantitative capability, clinical feasibility, and cost.

Unenhanced CT performed for other indications can give a rough estimate of hepatic steatosis via reduced liver attenuation; however, this method is not suitable for routine screening or accurate assessment [4]. In a recent systematic review, CT—limited to the perirenal and RSF compartments—showed higher RSF in diabetes and obesity, with no reliable assessment of renal parenchyma fat [130].

For hepatic steatosis, MRI-PDFF is the reference standard imaging tool [129]. Also, magnetic resonance spectroscopy (MRS) is a reliable non-invasive alternative for quantifying fat and grading hepatic steatosis [131]. For the kidney, MRI can quantify parenchymal lipids, but methods are heterogeneous, and no clinical PDFF cut-offs exist; however, a provisional ≥4% has been suggested [130].

A concise rationale for why renal fat imaging currently lags behind hepatic imaging is summarized in Discussion, Section 7.2.

## 7. Discussion

### 7.1. The Hepatorenal Metabolic Axis

**Figure 2:**
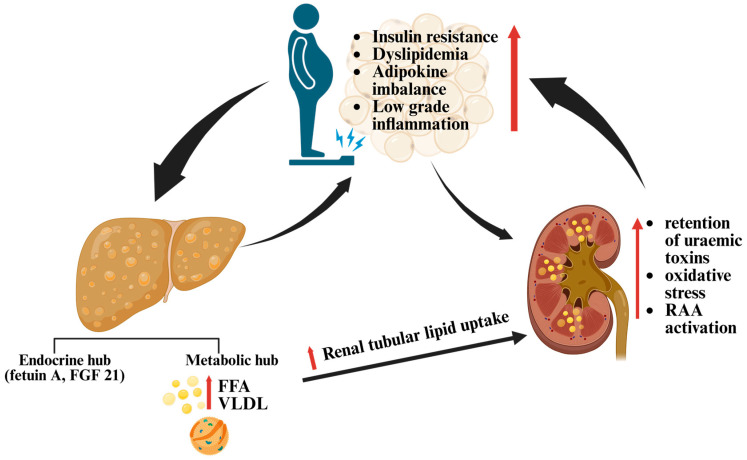
Schematic representation of the hepato–renal metabolic axis in metabolic dysfunction–associated steatotic liver disease (MASLD). Visceral adiposity, insulin resistance, dyslipidemia, adipokine imbalance, and low-grade inflammation establish a bidirectional crosstalk between the liver and kidney. The steatotic liver releases excess lipids and hepatokines that promote renal lipotoxicity, while renal dysfunction amplifies systemic inflammation and dysmetabolism, thereby aggravating hepatic steatosis. Black arrows indicate the direction of the interactions between visceral adiposity, the liver and the kidney within this hepato-renal metabolic axis, whereas red upward arrows denote increased levels of the respective processes or factors. (Created in BioRender. Ghiga, D. (2025) https://BioRender.com/2c9ec31).

A schematic representation of the liver-kidney axis and its metabolic mediators is shown in Figure 2.

Visceral adiposity and metabolic dysfunction—insulin resistance, dyslipidemia, adipokine imbalance, and low-grade inflammation—establish a bidirectional liver–kidney crosstalk in MASLD. The steatotic liver acts as an endocrine hub by releasing hepatokines (notably fetuin-A and FGF21) [92], but also as a metabolic hub determining excess free fatty acids, VLDL. This increased lipid output drives renal tubular lipid uptake, mitochondrial/oxidative stress, and macrophage-driven inflammation, and accelerates lipotoxic fibrosis [35]. Conversely, as kidney function declines, retention of uraemic toxins, oxidative stress, and renin–angiotensin–aldosterone activation aggravate insulin resistance, dyslipidemia, and systemic inflammation, worsening hepatic steatosis and promoting fibrogenesis [18]. Mechanistically, both organs conform to a shared multi-hit model: renal lipid excess becomes pathogenic when coupled with inflammatory signaling and mitochondrial dysfunction—precisely the pattern described by Su et al., which mirrors the multi-factorial pathogenesis of steatotic liver disease [132].

Within this hepatorenal axis, imaging-detected renal ectopic fat can be viewed as the kidney-side counterpart of hepatic steatosis. It may occur across the MASLD spectrum (including lean MASLD) within the same adverse metabolic milieu.

### 7.2. Why Renal Fat Imaging Lags Behind Hepatic

Renal fat imaging currently lags behind hepatic imaging due to compartmentalization, low parenchymal signal, motion-related artifacts, lack of harmonized methods, and outcome-anchored thresholds. In radiology studies, what has been described as a fatty kidney phenotype has emerged as a renal counterpart of MASLD due to renal ectopic fat deposition [122], yet in routine care, it likely remains underreported because consensus definitions and outcome-anchored thresholds by compartment are missing.

Unlike diffuse hepatic steatosis, already quantifiable by various standardized tools [133], renal lipids are compartmentalized (perirenal, sinus, and a small cortical/medullary fraction). This renders the parenchymal PDFF/MRS signal intrinsically low and amplifies noise. Respiratory and vascular motion, together with the kidney`s layered corticomedullary anatomy, cause image misregistration and partial-volume effects, even with careful breath-holds.

Protocols and analyses remain non-harmonized across vendors, and there are no histology- or outcome-anchored thresholds for renal PDFF/MRS, which limits reproducibility and slows clinical adoption. These anatomic, standardization, and validation gaps—not the capability of any single modality—explain the slower translation compared with hepatic imaging. The prevalence and clinical impact of imaging-recognized renal steatosis in routine practice remain to be determined.

### 7.3. Clinical Implications

Population-based cohort data link RSF with hypertension and CKD independent of adiposity, supporting its use as a cardiometabolic risk marker [123].

In patients with T2DM, higher renal parenchymal PDFF correlated with greater DKD severity, suggesting that even small MRI-PDFF increases may be meaningful [134]. In another study, RSF was found to be higher in diabetes and to independently predict incident diabetes [135].

Beyond metabolic risk, renal fat has been associated with focal segmental glomerulosclerosis, obesity-associated glomerulopathy, nephrolithiasis, CKD, ESKD, and kidney cancer [136,137,138,139,140]. Because most links between renal fat and these outcomes come from obese and metabolically unhealthy cohorts with substantial confounding, it remains difficult to disentangle the contribution of renal fat per se from that of overall adiposity and metabolic risk. Paradoxically, in dialysis, obesity is associated with better survival [141].

In MASLD, kidney evaluation should not rely solely on creatinine. Incorporate albuminuria, consider simple imaging surrogates (perirenal/RSF where available), and track tubular stress markers as adjuncts.

These implications support targeting shared metabolic pathways with potential dual-organ benefit.

### 7.4. Emerging Therapeutic Convergence

Practical management rests on two pillars: sustained weight loss and pharmacotherapy with overlapping hepatorenal benefits.

#### 7.4.1. Lifestyle Interventions

The cornerstone for treating MASLD and simultaneously mitigating CKD risk remains sustained weight loss. The 2024 EASL–EASD–EASO guideline states that a ≥5% weight loss is needed to improve liver histology in MASLD. Mediterranean-inspired diet, reduced alcohol consumption, and aerobic exercise have been shown to improve liver enzymes and stiffness [4,142].

These lifestyle measures also benefit the kidney by improving blood pressure, insulin resistance, and atherogenic lipid profiles. They are additionally associated with lower albuminuria and a slower eGFR decline [9].

#### 7.4.2. Pharmacotherapy with Hepatorenal Benefits

Sodium-glucose cotransporter-2 (SGLT2) inhibitors and statins are situated at the core of CKD management by the KDIGO 2024 guideline. These agents slow eGFR decline and reduce cardiovascular events; evidence is emerging that they also confer hepatic benefits [9]. Suki et al. reviewed data from randomized trials and observational studies involving MASLD patients and concluded that SGLT2 inhibitor therapy reduces liver steatosis and improves non-invasive fibrosis markers [143].

Often prescribed for cardiovascular risk in almost all CKD patients, statin therapy not only lowers LDL cholesterol but has been demonstrated to be beneficial in MASLD treatment, improving liver enzyme levels and slowing progression to advanced liver fibrosis [144,145].

Agonists of the glucagon-like peptide-1 receptor (GLP-1 RAs), such as semaglutide, provide clinically meaningful weight loss and increasingly recognized dual-organ benefits. In ESSENCE, semaglutide 2.4 mg weekly achieved 62.9% resolution of steatohepatitis without fibrosis worsening and 36.8% ≥1 stage fibrosis improvement compared with 34.3% and 22.4% in the placebo group. Secondary endpoints showed improvements in lipids, blood pressure, and markers of systemic inflammation [146].

These hepatic benefits parallel renal gains: the FLOW trial demonstrated that semaglutide reduces clinically important kidney outcomes and cardiovascular death in T2DM with CKD [147].

The dual-incretin agonist, tirzepatide, mirrors the hepatic response pattern seen with GLP-1 receptor agonists, and post-hoc SURPASS-4 analyses signal renoprotection (slower eGFR decline, lower albuminuria, fewer kidney events) [148,149].

### 7.5. Research Gaps and Limitations

Clinical vocabulary remains split: Importantly, the term fatty kidney is used as a descriptive imaging phenotype of renal fat accumulation, whereas ORG is a specific histopathologic entity characterized by glomerular hypertrophy and sclerosis in obesity. Thus, this phenotype captures renal fat deposition detectable by imaging, while ORG reflects biopsy findings of glomerular injury. To our knowledge, there is no validated imaging case definition for ORG and no histologic case definition for this phenotype. The two constructs likely represent complementary facets of the same metabolic injury rather than mutually exclusive diseases and may coexist in the same patient.

No formal diagnostic criteria for the fatty kidney phenotype have been established to date. However, several studies have proposed illustrative thresholds for research purposes—such as high sex-specific percentiles of RSF on CT [123], or MRI-PDFF values around 4% to flag increased renal lipid content [130]. These values are pragmatic rather than prescriptive, highlighting the need for standardized, outcome-anchored definitions and cross-modality reproducibility.

Conceptually, the overlap between MASLD and this fatty kidney phenotype probably corresponds to what could be described as MASLD-related kidney injury. However, most existing imaging data on renal fat come from studies designed from a renal or general metabolic perspective, in which renal fat was assessed separately and hepatic steatosis was not systematically phenotyped. As a result, current evidence is not yet sufficient to formalize such a diagnostic category.

Large, non-diabetic, multi-ethnic MASLD cohorts are needed, with paired liver staging (biopsy or validated non-invasive tools) and kidney phenotyping (imaging plus tubular biomarkers), and with hard renal endpoints (eGFR slope, persistent albuminuria, ESKD).

Human data that confirm mitochondrial and mitophagy dysfunction in podocytes and proximal tubules under lipotoxic stress—explicitly paralleled with hepatic MASLD—remain limited.

Beyond current signals, trials designed for the hepatorenal question are still few; future studies should stage liver disease and pre-specify renal outcomes to test whether weight loss, SGLT2 inhibitors, GLP-1 RAs, statins, or other lipid-modulating agents change kidney trajectories in MASLD.

### 7.6. Future Perspectives

Imaging work should prioritize simple, reproducible tools for routine care with ultrasound-based surrogates where feasible. For MRI, centers should use similar scan settings and analysis so results are comparable.

There is a clear need for early biomarkers that detect lipotoxic kidney injury before classical signs, especially in non-diabetic MASLD. Future studies should use integrated hepatorenal designs that pair liver staging (biopsy or validated non-invasive tests) with renal imaging and biomarker profiling, with pre-specified renal outcomes.

We foresee a practical paired-organ model for MASLD clinics: non-invasive liver fibrosis staging plus a renal lipotoxic panel (albuminuria and tubular proteins, with lipidomics where feasible) and, when possible, compartment-specific renal fat. Validating this model against renal outcomes—and embedding it in trials of metabolic/anti-lipotoxic therapies—could move renal fat from a descriptive label to an operational risk category with clear management consequences.

## 8. Conclusions

MASLD is increasingly recognised as a systemic metabolic disease with a lipotoxic footprint involving both liver and kidney, although the nature of this liver–kidney relationship is still only partly understood. Throughout this review, we summarise evidence from epidemiology, mechanistic studies, imaging, biomarkers, and emerging therapies to show that kidney involvement often develops within the same adverse metabolic milieu as MASLD, rather than as an entirely separate disease process. To capture the full burden of MASLD beyond the liver, clinicians should not rely on creatinine alone and should instead pair liver staging with simple kidney evaluation—albuminuria and eGFR, and, where available, assessment of renal ectopic fat—to anchor renal risk.

## Figures and Tables

**Table 1:** Hepatorenal lipotoxic mechanisms and key biomarkers.

Mechanistic Module	Liver (Marker → Direction)	Kidney (Marker → Direction)
FA uptake	CD36 ↑ in hepatocytes/Kupffer/HSC; steatosis + inflammatory signaling [42,43,44,45,46].	CD36 ↑ in podocyte/tubule leads to FA influx, ROS, profibrotic signaling [74,75,76]
Stress/adaptation hepatokine	FGF21 ↑ with metabolic stress/MASLD severity [110,111]	FGF21 ↑ in CKD [114,115] experimental renoprotection [117,118,119,120,121]
Inflammatory hepatokine	Fetuin-A ↑ in MASLD [94,95]	uPTM-FetA ↑ in tubular injury [105,106,107]
Tubular stress/injury		KIM-1 ↑ (early proximal tubular injury) [83,84,85]
		NAG ↑ in obese patients [84]
		NGAL ↑ (AKI–CKD stress; limited lipotoxic specificity) [83,84,85]
FA-handling tubular stress		uL-FABP ↑ (early injury; precedes creatinine) [87,88]
Renal lipidomics		DAGs, LPCs, PCs, PEs, LPEs ↑ in DKD [89]

Notes: ↑—increase.

**Table 2:** Comparative overview of liver and kidney fat imaging modalities.

Modality	Principle	Assessable Compartment (s)	Quantitative Capability	Clinical Feasibility	Cost
B-mode US (liver)	Qualitative hepatic echogenity; posterior beam attenuation, blurred vessels/diaphragm	Liver parenchyma	No (qualitative)	Very high	Low
CAP	Ultrasound attenuation (dB/m)	Liver parenchyma	Surrogate (continuous)	High	Low–Med
UDFF (US)	Quantitative backscatter/speed-of-sound	Liver parenchyma	Yes (fat fraction, %)	Emerging	Low–Med
MRI-PDFF/MRS (liver)	Chemical-shift water–fat separation/proton spectroscopy	Liver parenchyma	Yes (reference standard)	Medium	High
B-mode US (kidney)	Cortical echogenicity vs. liver/spleen	Renal parenchyma (echogenity; not fat)	No	Not applicable	Low
US renal sinus area	2D planimetry in sinus	RSF	Semi-quantitative (area)	High	Low
CT (non-contrast, kidney)	Attenuation (HU) and area/volume segmentation	Perirenal & RSF	Semi-quantitative	High	Medium
MRI Dixon/IDEAL-IQ (kidney)	Water–fat separation (chemical-shift)	Perirenal/sinus; parenchymal (research)	Yes (research PDFF)	Low–Med	High
MRS (kidney)	Proton spectroscopy	Parenchymal (research)	Yes (research)	Low	High

## Data Availability

No new data were created in this study.

## Abbreviations

The following abbreviations are used in this manuscript:

AKI	Acute Kidney Injury
BMI	Body Mass Index
CAP	Controlled Attenuation Parameter
CD36	Cluster of Differentiation 36
CKD	Chronic Kidney Disease
CT	Computed Tomography
DAG	Diacylglycerol
DKD	Diabetic Kidney Disease
EASL	European Association for the Study of the Liver
ECM	Extracellular Matrix
ER	Endoplasmic Reticulum
ESKD	End-Stage Kidney Disease
eGFR	Estimated Glomerular Filtration Rate
FFA	Free Fatty Acids
FGF21	Fibroblast Growth Factor 21
GLP-1 RAs	Agonists of the glucagon-like peptide-1 receptor
GLUT4	Glucose Transporter Type 4
IDEAL	Iterative Decomposition of water and fat with Echo Asymmetry and Least-squares estimation (MRI sequence)
IL-1β	Interleukin-1 beta
IL-6	Interleukin-6
IL-18	Interleukin-18
IR	Insulin Resistance
KDIGO	Kidney Disease: Improving Global Outcomes
KIM-1	Kidney Injury Molecule-1
L-FABP	Liver-type Fatty Acid-Binding Protein
LDL	Low-Density Lipoprotein
MASLD	Metabolic Dysfunction-Associated Steatotic Liver Disease
MASH	Metabolic Dysfunction-Associated Steatohepatitis
MRI	Magnetic Resonance Imaging
NAFLD	Non-Alcoholic Fatty Liver Disease
NGAL	Neutrophil Gelatinase-Associated Lipocalin
NLRP3	NOD-Like Receptor Protein 3 (inflammasome)
Ox-LDL	Oxidized Low-Density Lipoprotein
PNPLA3	Patatin-Like Phospholipase Domain-Containing 3
ROS	Reactive Oxygen Species
RSF	Renal Sinus Fat
SGLT2	Sodium-glucose cotransporter-2
T2DM	Type 2 Diabetes Mellitus
TAG	Triacylglycerol
TLR4	Toll-Like Receptor 4
TNF-α	Tumor Necrosis Factor Alpha
UDFF	Ultrasound-Derived Fat Fraction
UPR	Unfolded Protein Response
uL-FABP	Urinary Liver-Type Fatty Acid-Binding Protein
uPTM-FetA	Urinary Post-Translationally Modified Fetuin-A
VLDL	Very-Low-Density Lipoprotein
